# The combined effects of ultrasound and lactic acid in inactivating microorganisms on fresh radish (*Raphanus raphanistrum* subsp. *sativus*): Microbiological and quality changes

**DOI:** 10.1002/fsn3.1287

**Published:** 2019-11-28

**Authors:** Seyed Mohammad Bagher Hashemi, Khadijeh Abhari, Amin Mousavi Khaneghah

**Affiliations:** ^1^ Department of Food Science and Technology College of Agriculture Fasa University Fasa Iran; ^2^ Department of Food Science and Technology National Nutrition and Food Technology Research Institute Faculty of Nutrition Sciences and Food Technology Shahid Beheshti University of Medical Sciences Tehran Iran; ^3^ Department of Food Science Faculty of Food Engineering University of Campinas (UNICAMP) Campinas Brazil

**Keywords:** lactic acid, pathogen, radish, ultrasound

## Abstract

In order to reduce the risk of microbial contamination in fresh radish (Raphanus raphanistrum) and ensure its safety, combined effects of ultrasound and lactic acid in inactivating microorganisms and quality changes of radish were studied. Fresh radish samples were inoculated with *Listeria monocytogenes*, *Escherichia coli*, *Staphylococcus aureus*, and *Shigella sonnei* separately and were treated with lactic acid (L) 1% and 2%, ultrasound (U) with the amplitude of 0%, 25%, 50%, and 75% for 15 and 30 min and their combination. The quality parameters, including total phenol content, firmness, and total color change, were evaluated on the day of the experiment and after 24 hr of cold storage. Results showed that both applied treatments and their combinations had significant (*p* < .05) inhibitory effect on all of the studied bacteria. Total phenolic content of the ultrasound treated samples led to higher amounts comparing to other samples. Results showed that using ultrasound power (75%), for 30 min significantly (*p* < .05) decreased the firmness of samples after 24 hr of cold storage. In conclusion, the application of ultrasound and lactic acid can extend the shelf life of fresh radish.

## Graphical abstract

Radish is known as a root vegetable used in treatment of many diseases. Radish can be contaminated with foodborne pathogens. Combination of lactic acid and ultrasonic showed the best results. Ultrasound along with lactic acid in can be considered new preservation technology.

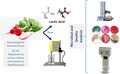

## 1 INTRODUCTION

Radish (*Raphanus sativus*) has been known as a root vegetable that is cultivated and consumed in many parts of the world. Although this vegetable is considered as a part of daily meals in some parts of the world, it is unpopular among some people. The common form of its consumption is raw and crunchy (Banihani, 2017). Radish has been recognized as a household treatment in many diseases such as liver diseases, gall bladder stone, jaundice, rectal prolapse, dyspepsia, and other gastric pains since long time ago in Unani, Greeko‐Arab, and India (Jeong et al., 2005; Shukla et al., 2011). Radish contains water‐soluble vitamins (B1, B2, B3, B5, B6, B9, and C), carbohydrates, dietary fibers, minerals (calcium, iron, magnesium, manganese, zinc, potassium, and phosphorous), protein, sugars, fluoride, and even some fat (Baenas et al., 2016; Ishida et al., 2015; Khattak, 2011; Malik, Riley, Norsworthy, & Bridges, 2010). According to the studies, the polyphenolic content of radish was assessed to be in the range of approximately 13.18–63.54 mgg^−1^ dry weight.

Nowadays, consumers prefer to include fresh vegetables in their daily diet due to their awareness about the health benefits and importance of vegetables due to their significant amount of vitamins, minerals, and dietary fibers of them. Moreover, consuming vegetables can reduce the risk of various diseases particularly cancer as several investigations indicated the correlation between health‐promoting phytochemical components in cruciferous sprouts such as broccoli and radish such as glucosinolates and cancer prevention (Jovanović, Klaus, & Nikšić, 2016; Martínez‐Villaluenga, Frías, Gulewicz, Gulewicz, & Vidal‐Valverde, 2008). The challenging issue is that the vegetables can be contaminated with foodborne pathogens during cultivation, growth, harvesting, transport, and distribution. The main sources of contamination are animal excretions, untreated irrigation water, surfaces of transportation trailers, and hands of handlers (Bang et al., 2016; Jovanović et al., 2016; Zhang, Cao, Hung, & Li, 2016).

Foodborne bacteria are the main cause of human diseases transmitted from foodstuff. Therefore, any preventive approach in case of destroying or lowering the amount of these pathogens is of high importance. According to the US Center for Disease Control and Prevention, the number of 48 million foodborne illnesses was estimated in the United States every year, of which 128,839 lead to hospitalizations and nearly 3,037 cases of deaths (Sugrue, Tobin, Ross, Stanton, & Hill, 2019). Foodborne diseases are mainly caused by the consumption of spoiled food by pathogens or their toxins and are easily spread, and therefore, result in worldwide public health problems. Foodborne pathogens contaminate food in different stages of production and delivery. There are many pathogens that are thermostable and show resistance to typical food preparation methods (cooking, frying, and freezing). Therefore, there should be other new strong approaches to get away with these microorganisms (Martinović, Andjelković, Šrajer Gajdošik, Rešetar, & Josić, 2016).

Lactic acid has been known as a novel food preservative to prolong the shelf‐life of food products (Noori, Khanzadi, Fazlara, Najafzadehvarzi, & Azizzadeh, 2018). It has the potential for suppressing microbial growth and prevents the production of microbial metabolites such as biogenic amines in many different food models (Noori et al., 2018).

Considering the ever‐increasing consumer preference to ready to use fresh products, and since the conventional preservative methods are associated with nonreversible changes in the foodstuff, e.g., thermal processing, today, a great majority of scientists are directing their research works toward novel nonthermal methods to minimize the process quality changes and in the same way to raise product shelf life (Birmpa, Sfika, & Vantarakis, 2013; Cao et al., 2010). Among the mentioned nonthermal processing techniques, ultrasound power as an environmentally safe and environment‐friendly technique has gained great interest during the last decade in many branches of scientific works. Ultrasound power, states sound waves of 20 kHz to the 100 kHz frequency that makes changes in the physical and chemical properties of biological systems mainly due to the cavitation principle (Hashemi, 2018), followed by reducing in the processing time and increasing its efficiency (Hashemi, Michiels, Yousefabad, & Hosseini, 2015). The effect of sonication power on microorganism inactivation in products has been studied by many researchers (Birmpa et al., 2013; Cao et al., 2010; Wang, Hu, & Wang, 2010), e.g. Birmpa et al. (2013) investigated its effect on *Salmonella Enteritidis*, *Listeria innocua*, *Escherichia coli*, and *Staphylococcus aureus*. However, the combined effect of ultrasound and lactic acid on microbial inactivation of radish has not so far been reported. Therefore, this study was aimed to investigate the combined effect of ultrasound power and the lactic acid at different powers and concentrations in inactivating four major foodborne pathogens and their effects on physicochemical properties in radish (Birmpa et al., 2013).

## 2 MATERIALS AND METHODS

### 2.1 Radish sample

Fresh radishes were purchased from a local store (Shiraz) on the day of the experiment and stored at 4°C for 2 hr until the time of analysis.

### 2.2 Bacterial strains

*Bacterial strains include Escherichia coli* PTCC 1,399, *Staphylococcus aureus* subsp. *aureus* PTCC 1764, *Listeria monocytogenes* PTCC 1,299, and *Shigella sonnei *PTCC 1777 were obtained from the culture collection of Iran Institute of Industrial and Scientific Research (Tehran, Iran). Stock cultures were inoculated into 25 ml of soybean casein digest medium USP (Oxoid, UK; CM1065) separately and incubated at 37°C for 18 hr. The culture was centrifuged at 4,260 *g* for 15 min at 4°C and washed with buffered peptone water. The final pellets were re‐suspended in buffered peptone water to give a population of log 7.1–7.6 CFU/ml.

### 2.3 Sample inoculation

Radishes were washed with running deionized water and left to dry in a laminar flow hood for 30 min. The prepared radishes were manually cut into wedges (10 ± 0.2 g) with a sterile knife. To inoculate the bacterial strains, 100 μl of each bacterial suspension was spotted on the surface of radish samples separately. For control samples, 100 μl of deionized sterile water was used. The inoculated samples were air‐dried at 25°C for 3 hr to allow bacterial attachment.

### 2.4 Sample treatment

**Figure 1:**
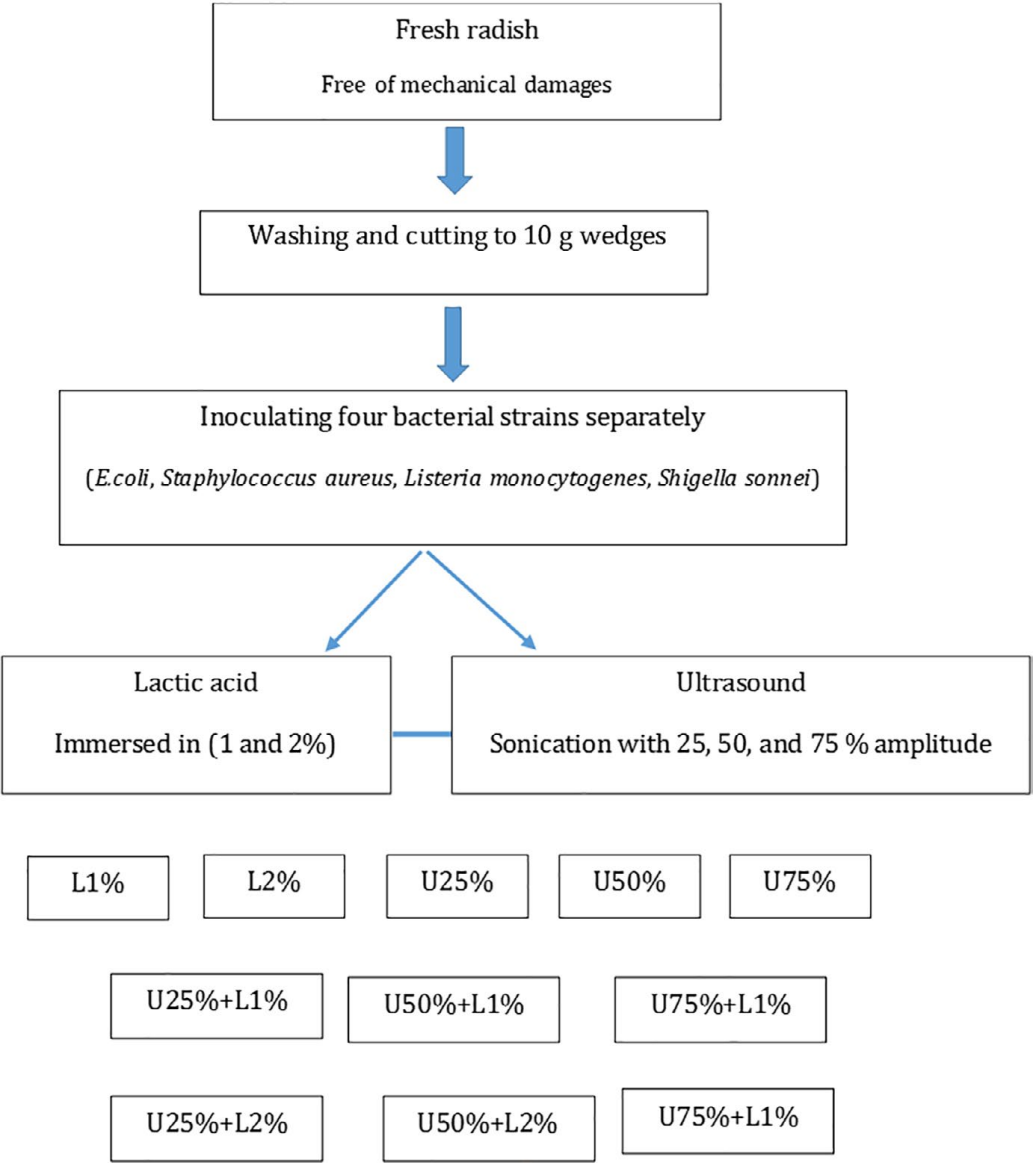
Flowchart of different treatments on radish

Twelve groups were designed for different treatments using lactic acid and ultrasound (Figure 1). For lactic acid treatments (L), 10 g of inoculated radish samples were immersed in 500 ml of lactic acid 1% or 2% (v/v) solutions at 25°C. Ultrasonication was performed by ultrasound (Hielscher ultrasonic device; UP100H, Germany; 100 W, 30 kHz) with a titanium sonotrode (tip diameter 10 mm) at 25%, 50%, and 75% amplitude levels. Sonication treatment was carried out on the sample by inserting the probe about 4 cm from the top into the cell. Each treatment was performed for a duration of 15, and 30 min and microbial analysis was carried out after various treating. To evaluate the changes in total phenolic compounds, firmness, and color, the treated samples were stored for 24 hr in 4°C (85%–90% RH). All the treatments were prepared for triplicates.

### 2.5 Microbial analysis

For microbial analysis, the samples were transferred into a sterile stomacher bag and homogenized with peptone water (0.1%) for 5 min with a stomacher (BagMixer 400 W, Interscience Co.). Following serial dilutions, 0.1 ml of diluents were spread plated onto each proper medium. MacConkey agar (PO0232, Oxoid, UK), Baird‐parker agar (PO0168, Oxoid, UK), Brain heart infusion agar (CM1135, Oxoid, UK), and Nutrient agar (CM0003, Oxoid, UK) were applied to enumerate *E. coli*, *S. aureus subsp. aureus*, *L. monocytogenes,* and *S. sonnei*, respectively. Afterward, plates were incubated at 37°C for 24–48 hr. As the four mentioned pathogenic bacteria were inoculated separately on different sterile radish samples, each sample contained only one of the inoculated bacteria and the target grown colonies were counted on selective mediums (Hashemi, 2018).

### 2.6 Determination of texture and color

Texture and color analysis was carried out on day 0 and 1. A texture analyzer (Lloyd, TA plus Instruments Ltd.) was applied to perform firmness analysis using a 5 mm diameter cylinder probe. Changes in the color of samples were measured with a Chroma meter (Chroma Meter model CR‐410; Minolta Co. Ltd.). To calculate the color change (∆*E*) the following equation was used:

(1) $$\Delta E=\sqrt{{\left(L_{0}-L_{t}\right)}^{2}+{\left(a_{0}-a_{t}\right)}^{2}}+{\left(b_{0}-a_{t}\right)}^{2}$$

The initial color of radish is displayed as *L*_0_, *a*_0_ and *b*_0_, and *L_t_*, *a_t_* and *b_t_* are demonstrating the color at specific times (Hashemi, 2018).

### 2.7 Measurement of total phenolic compounds

For measurement of total phenolic compounds, radish samples were extracted (Mechanical juicer; Pars Khazar) and centrifuged (EBA21, Hettich) at 3,000 *g* for 10 min. Then, 100 µl of supernatant was blended with 500 µl of 1:10 diluted Folin–Ciocalteu reagent. After adding 400 µl of sodium carbonate, incubation was performed at room temperature for 30 min, followed by measurement of absorbance at 765 nm using spectrophotometer (Philips) (Gheisari & Abhari, 2014; Stintzing et al., 2005).

### 2.8 Statistical analysis

The analysis was done in triplicate and data expressed as mean values of three repetitions followed by the standard deviation. One‐way ANOVA was used to compare the means at the significance level of *p* < .05, and Duncan's multiple range test was performed to differentiate the treatment means. All statistical analyses were done using the SPSS package program 20 (SPSS 20.0 for Windows; SPSS Inc.).

## 3 RESULTS AND DISCUSSION

### 3.1 Microbial inactivation in radish by lactic acid and ultrasound power

**Figure 2:**
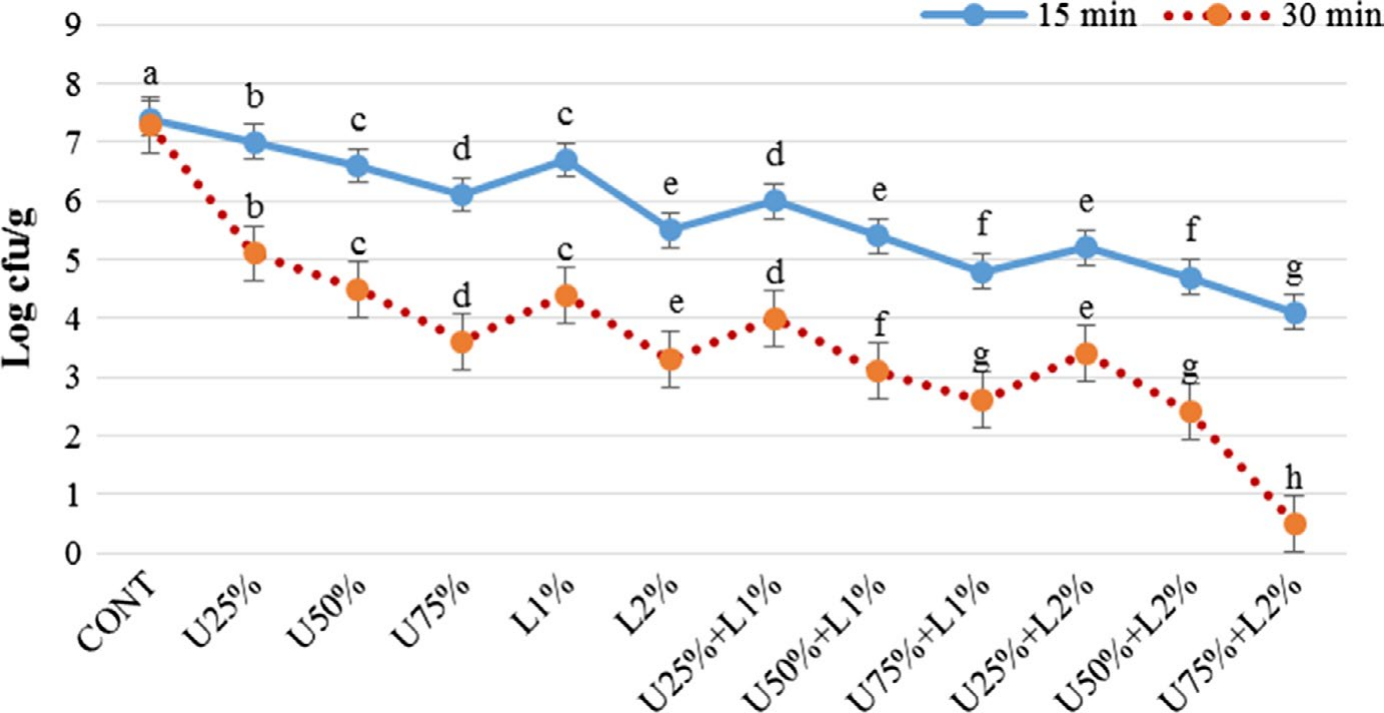
Effect of lactic acid and ultrasonic power and their combination on number of *E. coli* in radish. In the same treatment time, bars not noted by same letter are significantly different (*p* < .05). Bars represent standard deviations

**Figure 3:**
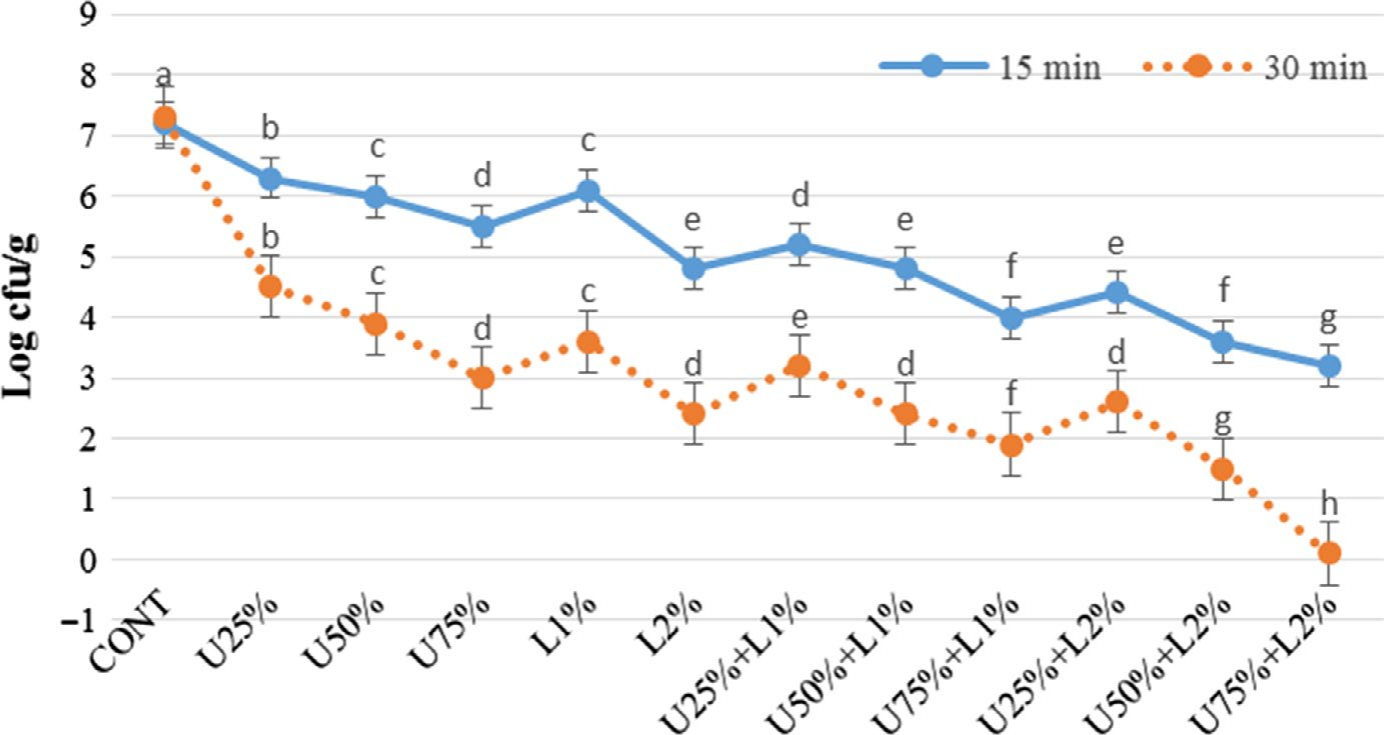
Effect of lactic acid and ultrasonic power and their combination on number of *Shigella sonnei* in radish. In the same treatment time, bars not noted by same letter are significantly different (*p* < .05). Bars represent standard deviations

**Figure 4:**
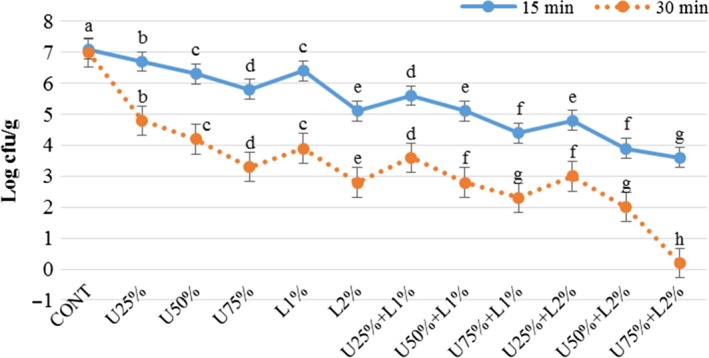
Effect of lactic acid and ultrasonic power and their combination on number of *Staphylococcus aureus* in radish. In the same treatment time, bars not noted by same letter are significantly different (*p* < .05). Bars represent standard deviations

**Figure 5:**
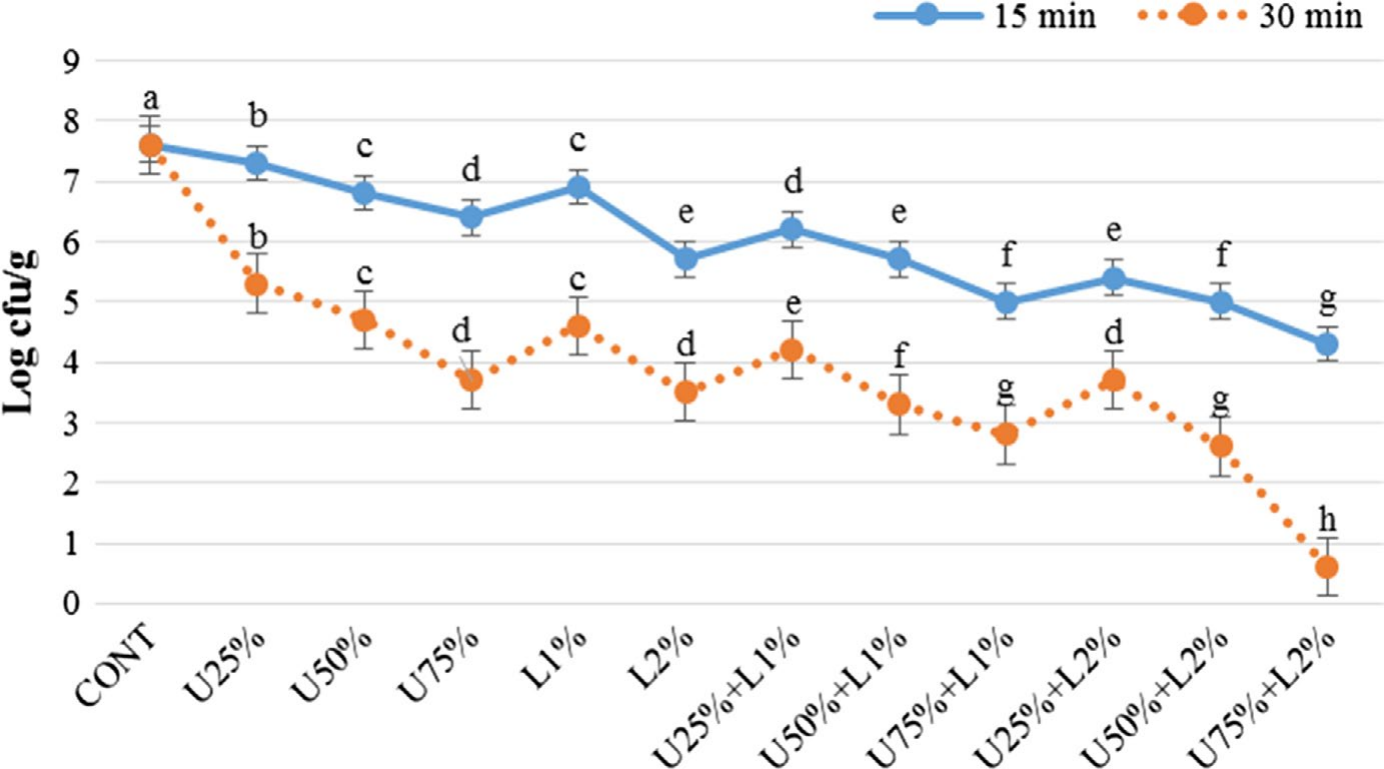
Effect of lactic acid and ultrasonic power and their combination on number of *Listeria monocytogenes* in radish. In the same treatment time, bars not noted by same letter are significantly different (*p* < .05). Bars represent standard deviations

The effect of lactic acid, ultrasound power, and their combination on *Listeria monocytogenes, Escherichia coli, Staphylococcus aureus,* and *Shigella sonnei* are presented in Figures 2, 3, 4, 5. According to the obtained results, both applied treatments include lactic acid and ultrasound power, and their combinations had a significant inhibitory effect on all of the studied bacteria. Generally, lactic acid was more effective in the reduction of inoculated pathogen compared to ultrasound power, and as it was expected an increase in the concentration of lactic acid or amplitude of ultrasound lead more reduction in the bacterial count. Comparing the count of four microorganisms shows a combination of lactic acid and ultrasound was more effective (*p* < .05) compared to treatments that applied them solely. A combination of lactic acid 2% and ultrasound with 75% amplitude caused 3 and 7 log reduction in the count of the inoculated pathogen microorganisms after 15 and 30 min, respectively. In treatment U75% + L2%, the initial level of *E.coli* and *Listeria monocytogenes* (log 7.4 CFU/g) was reached to around log 4.2 and 0.5 CFU/g after 15 and 30 min of treating, respectively. This treatment was also more effective on *Staphylococcus aureus* and *Shigella sonnei,* which reached nearby log 3.3 and 0.2 CFU/g after 15 and 30 min, respectively. As it is demonstrated in the Figures 2, 3, 4, 5, prolonging the exposure to different treatments resulted in a decrease (*p* < .05) in the count of all bacteria, hence treating for 30 min lead to more reduction in all of the studied microorganisms compared to 15 min. The effect of lactic acid on the extension of the microbiological shelf life has been reported in many investigations (Noori et al., 2018). Lactic acid is considered as a strong anti‐bacterial agent, especially as the pH reaches to pKa of 3.86, due to free lactic acid disseminating through cellular membranes (Miller, 2017). Ultrasound cavitation also is known to be the main mechanism of microbial inactivation, which corresponds to the bubble production through continuous cycles of bursts which lead to forming hot spots and increase in pressure and temperature, which may probably lead to the disruption of cell membranes, creating chemical reactions, principally free radicals (Birmpa et al., 2013; Hashemi, 2018; Jambrak, Lelas, Mason, Krešić, & Badanjak, 2009). Hashemi (2018), studied the effect of pulsed ultrasound treatment on Mirabelle plum during postharvest storage and found that ultrasound treatment was more effective in reducing the total count of microorganisms (*p* < .05) and the quality was improved during storage time. They indicated lactic acid was more effective than ultrasound treatment, even at its minimum concentration of 1%. Thus it was concluded its application is much more reasonable compared to the application of lower amplitudes of ultrasound (Hashemi, 2018).

It is known that the efficacy of power ultrasound in food systems depends on many factors. The factors related to ultrasound treatment such as the amplitude of sonication, temperature, time, volume, food composition, type of targeted microorganism, and initial microbial counts are the factors that should be considered in applying ultrasound as a decontamination method. Two forms of ultrasonication procedure are available to use in food matrices based on the used equipment: ultrasonication via bath or probe. Each method has its own advantages and limitations and is appreciate for specific conditions and food. Ultrasonication with probes is worked by inserting a rod‐shaped metal probe in the medium or food matrix. This kind of ultrasonication is more intensive while compared to bath ultrasonication. However, when using this system elevating the temperature in the food samples and its consequences should take into consideration (de São José et al., 2014).

Regarding the microbial inactivation, different sensitivities can be expected based on the size, shape, and species of the microorganisms. The previous studies indicated that bigger cells are more sensitive to ultrasonication (Gracin et al., 2016).

In the current study, a synergetic effect was observed between lactic acid and sonication which can be explained as ultrasound had both physical and chemical effects through generating bubbles which can cause cavitation and finally collapse cell and creation of free radicals. These damage to cell membranes provide the condition for lactic acid to penetrate the microorganisms and cause cell death.

### 3.2 Effect of lactic acid and ultrasound power on the total phenol content (TPC) of radish

**Figure 6:**
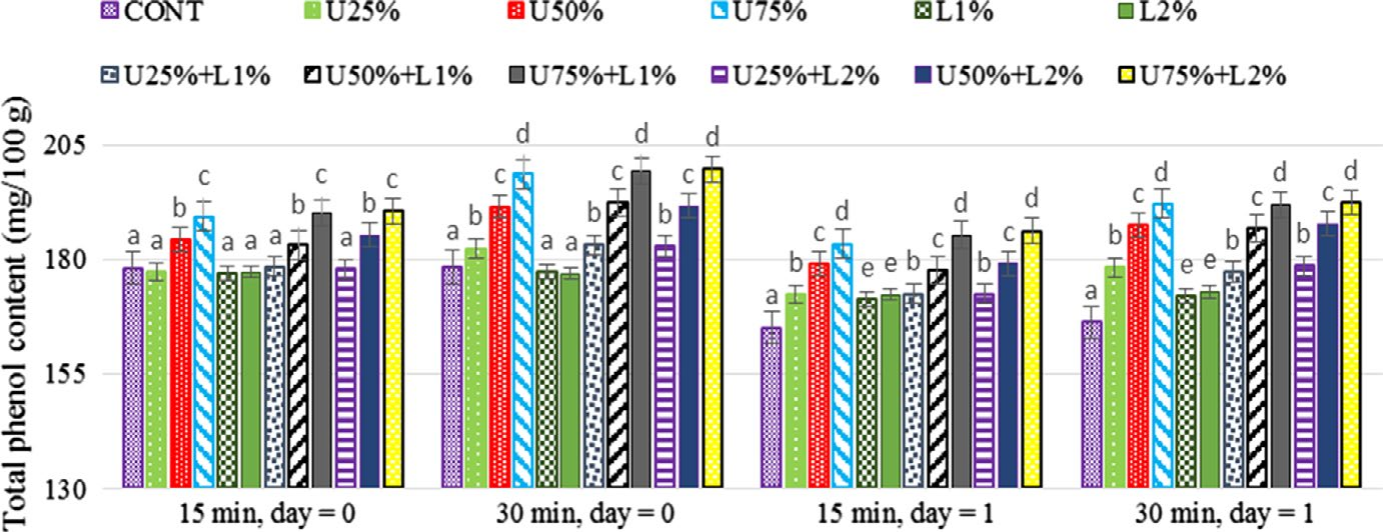
Effect of lactic acid and ultrasonic power and their combination on total phenolic content (mg/100 g) in radish. In the same treatment time and day, bars not noted by same letter are significantly different (*p* < .05). Bars represent standard deviations

The effect of lactic acid, ultrasound power, and their combination on the total phenolic content (mg/100 g) in radish was investigated on days 0 and 1 (Figure 6). Results showed that storing radishes (at 4°C) for 24 hr has led to a significant (*p* < .05) decrease in the amount of the total phenolic content. Many studies indicated that exposure to light and oxygen causes a substantial reduction in phenolic compounds and antioxidant activity due to their sensitivity and instability (Ali et al., 2018). Based on the results, the total amount of phenolic compounds in ultrasound treated samples was higher (*p* < .05) comparing to lactic acid‐treated and control samples which were mainly due to the mechanical consequence of the cavitation phenomenon, which damage the wall matrix leads to release of intracellular content, e.g., phenolic compounds (Sumere et al., 2018). The obtained results in the present study are in agreement with Hashemi (2018), who applied ultrasound on Mirabelle plum and stated ultrasonication caused an increase in total phenolics of fruit samples. Elevating in total phenol contents of purple cactus pear juice and red raspberry puree, treated with ultrasound also was declared (Golmohamadi, Möller, Powers, & Nindo, 2013; Hashemi, 2018; Zafra‐Rojas et al., 2013).

Golmohamadi et al. (2013), explained that the ultrasound process causes an increase in temperature which leads to enhance the leakage of phenolic compounds from the cells. On the contrary, the application of lactic acid had no significant (*p* > .05) effect on the total phenol content of radish, which may be due to its inability to extract the phenolic compounds.

### 3.3 Effect of lactic acid and ultrasound power on the firmness of radish

**Figure 7:**
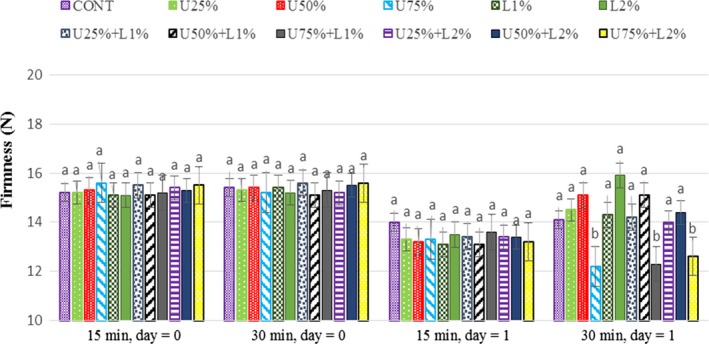
Effect of lactic acid and ultrasonic power and their combination on firmness (*N*) in radish. In the same treatment time and day, bars not noted by same letter are significantly different (*p* < .05). Bars represent standard deviations

The firmness results of radish samples are displayed in Figure 7. There were no significant differences (*p* > .05) in the firmness of treated samples on day 0 compared with control. Firmness results showed that using ultrasound power with an amplitude of 75%, for 30 min significantly (*p* < .05) decreased the firmness of samples after 24 hr of cold storage. The effect of high power (75%) ultrasound treatment on the texture of samples during storage can be a result of cell wall degradation, releasing the enzymes and consequently softness of the texture (Cao, Hu, & Pang, 2010; Cao et al., 2010; Chen & Zhu, 2011; Hashemi, 2018).

### 3.4 Effect of lactic acid and ultrasound power on the total color change of radish

**Figure 8:**
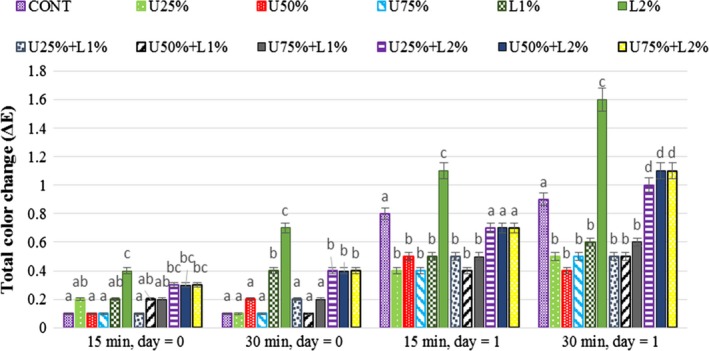
Effect of lactic acid and ultrasonic power and their combination on total color change in radish. In the same treatment time and day, bars not noted by same letter are significantly different (*p* < .05). Bars represent standard deviations

Total color change (Δ*E*) of the studied samples is presented in Figure 8. The total color change did not show any significant difference (*p* > .05) between the treated samples on the day 0, however storing the samples for 24 hr caused a significant color change in all treated samples and control. It can conclude from the results that applying lactic acid 2% was the most cause of color change in the samples either alone or in combination with an ultrasound. The results also display that the samples treated with lactic acid (2%) and ultrasound power at the highest applied level (75%) were found to be the most undesirable samples due to their dark color. However, the studied storage time (1 day) was not enough to come to a precise conclusion about the effect of each treatment and the appropriate level and duration. It seems that the control sample was recognized to be more suitable than the treated samples in case of color change. May ultrasonication treatment produces new reflective angles through the cavitation process, which in turn leads to the darkness of the samples. Another explanation may be due to the browning reactions that result in higher total color changes and represent the quality loss of the product. The marketing of these fresh‐cut radishes is limited due to their short shelf‐life and fast deterioration of their ingredients mainly because of tissue damage by slicing (Watada, 1997). Slicing would accelerate biological changes such as enzymatic browning (Ahvenainen, 1996).

## 4 CONCLUSION

In the current study effect of lactic acid in a concentration of 1% and 2%, ultrasound power with an amplitude of 25%, 50% and 75% and their combination on four inoculated pathogens and quality of fresh radish was investigated. Ultrasound treatment has shown promising results along with the application of lactic acid in reducing total microbial count and improving total phenolic content and firmness of the samples. This shows that investments in new preservation technologies are being improved by gaining enough favorable outcomes over applying these procedures. The ultrasound treatment has proved its role in keeping the fresh quality of foodstuff through storage time, which is an indispensable quality criterion in the food preservation area. In conclusion, it seems quite necessary to consume minimally processed fruits and vegetables to confirm their nutritive value toward using them.

## CONFLICT OF INTEREST

The authors declare that they have no competing interests.

## ETHICAL APPROVAL

The human and animal testing was unnecessary in the current study.
